# Gastroduodenal lesions in upper gastrointestinal endoscopy associated with positive *Helicobacter pylori* histology in cirrhotic patients at Sikasso Hospital: prevalence study

**DOI:** 10.11604/pamj.2020.37.123.20014

**Published:** 2020-10-05

**Authors:** Oumar Traoré, Abdoul Salam Diarra, Oumar Kassogué, Tawfiq Abu, Saidou Touré, Aguissa Maïga, Moussa Diarra

**Affiliations:** 1 Service of Gastro-Enterology, Regional Hospital of Sikasso, Sikasso, Mali,; 2 Reproductive Health Division, Regional Health Direction, Mopti, Mali,; 3 Service of Laboratory, Blood Bank, Regional Hospital of Sikasso, Sikasso, Mali,; 4 Department of Urology, Hassan II University Hospital Center, Fez, Morocco,; 5 Service of Internal Medicine, Regional Hospital of Sikasso, Sikasso, Mali,; 6 Administration Division, Regional Health Direction, Mopti, Mali,; 7 Service of Gastro-Enterology, Gabriel Touré University Hospital, Bamako, Mali

**Keywords:** Gastroduodenal lesions, *Helicobacter pylori*, cirrhosis, histology, digestive endoscopy

## Abstract

The presence of Helicobacter pylori is a major contributor to the genesis of peptic ulcer disease, although its role in the pathogenesis of ulcer in cirrhotic patients is yet to be well established. The aim of this work is to determine the prevalence of gastroduodenal lesions associated with histologically confirmed Helicobacter pylori in cirrhotic patients. This was a retrospective study which was conducted from January 2017 to May 2018 at Sikasso Hospital. The inclusion criteria were: presence of cirrhosis, endoscopic gastroduodenal lesions for which histological confirmation of the presence of Helicobacter pylori biopsies was made. The collected data was analyzed by Epi Info software version 7.0. Thirty four patients have been included, the mean age was 38 ± 17 years and a male/female sex ratio of 2.09. Gastrointestinal symptoms included epigastralgia (26.47%), nausea (8.82%), early postprandial vomiting (5.88%) and hematemesis (8.82%). Esophagogastroduodenoscopy revealed esophageal varices in 47%, which 1 case of esophageal varices grade III with red signs, 5.88% grade II with red signs, 8.82% grade I without red signs. A case of portal hypertension gastropathy was noted in 12 patients and gastroduodenal lesions in 33%. Anatomopathological examination of the biopsies revealed Helicobacter pylori in 57%, active chronic gastritis in 44.11% and chronic gastritis with intestinal metaplasia in 2.94% of cases. This study reveals a fairly high frequency of Helicobacter pylori in digestive lesions observed in cirrhotic patients. Helicobacter pylori infection in cirrhotic patients requires urgent therapeutic management to prevent the possible hemorrhagic complications.

## Introduction

Cirrhosis is the ultimate stage of all chronic liver diseases and as a result it causes enormous complications, some of which are life-threatening in the short and medium term. Upper gastrointestinal bleeding is one of the most dangerous complications in cirrhotic patients. Among the main causes of these hemorrhages in cirrhotic patients are: rupture of esophageal varices which is the most frequent (57.4%); followed by gastric ulcers (13.9%) and duodenal ulcers (11.1%) [1]. The prevention of these hemorrhages involves searching for all causes such as digestive lesions, in particular gastroesophageal varices and gastric ulcers likely to cause bleeding. Hemodynamic changes in cirrhotic patients lead to significant weakening of the gastroduodenal mucosa, which causes certain disorders such as those of its vascularization. The presence of *Helicobacter pylori* is a major contributor to the genesis of peptic ulcer disease, although its role in the pathogenesis of ulcer in cirrhotic patients has not yet been well established. The objective of this study was to determine the prevalence of gastroduodenal lesions associated with positive histological *Helicobacter pylori* in cirrhotic patients at the Sikasso Regional Hospital.

## Methods

It was a retrospective study of cirrhotic patients due to various etiologies, collected from January 2017 to May 2018 at Sikasso Hospital. All patients included in this study underwent esophagogastroduodenoscopy (EGD) during which two antral biopsies, two fundic biopsies and one angulus biopsy were performed. A histological study of the samples taken was performed at the pathology department of Bamako to confirm the presence of *Helicobacter pylori*. The gastroduodenal ulcers objectified by the EGD were classified according to the FORREST classification. Non-cirrhotic patients, those who did not perform EGD, as well as patients whose histological examination of the biopsies were negative for *Helicobacter pylori*, were not included in this study. The data were collected from the endoscopy and results of the anatomy-pathology examinations requested registry. The data was analyzed using Epi Info software version 7.0. All quantitative variables were described in terms of mean plus standard deviation and qualitative variables in terms of percentage.

## Results

A total of 34 patients were used in our study. The average age was 38 ± 17 with extremes of 16-81 years and a male predominance (67.6%). In our case study patients, there were post hepatitis viral B cirrhosis in 21 patients, cirrhosis due to viral hepatitis C in 8 patients, post-ethyl cirrhosis in 1 patient, primary biliary cirrhosis in 1 patient, and cryptogenic cirrhosis in 3 patients. Clinically, a total of 17 patients (50%) had gastrointestinal symptoms; epigastralgia (26.47%), nausea (8.82%), early postprandial vomiting (5.88%) and hematemesis (8.82%). The esophagogastro duodenoscopy revealed gastroesophageal varices in 47% (16 cases), of which 11.76% (4 cases) were esophageal varices grade III without red signs, 1 case of esophageal varices grade III with red signs, 5 cases (14 cases), 70% of esophageal varices grade II without red signs, 2 cases (5.88%) of esophageal varices grade II with red signs, 3 cases (8.82%) of esophageal varices grade I without red signs and 1 case of oesogastric varices (GOV) grade 1. Portal hypertension gastropathy was noted in 12 patients (35.29%), gastroduodenal lesions in 33% of which 17.64% were erythematous antro-fundic gastritis, 2 cases (5.88%) of antral gastric ulcers and 3 cases (8.82%) of antral ulcerations. Peptic oesophagitis was present in 20% of cases, a simple bulbitis in 13% of cases, an uncomplicated hiatal hernia in 5.88% of cases. Using the FORREST classification, stage III gastroduodenal ulcers were the most recorded. Anatomopathological examination of the biopsies carried out revealed *Helicobacter pylori* in 57% of cases, active chronic gastritis in 44.11% (15 cases) and chronic gastritis with intestinal metaplasia in 1 case (2.94%). Management of all lesions observed was done at Sikasso Hospital.

## Discussion

The principal esogastroduodenal lesions observed during digestive endoscopy in cirrhotic patients constitute a deleterious prognostic factor because they can cause digestive hemorrhages that can lead to short life terms in these patients. *Helicobacter pylori*, known as the most incriminated bacterium in digestive tract lesions, requires special attention in cirrhotic patients. It is with the aim of providing early, adapted and effective management in cirrhotic patients, we conducted this study to determine the frequency of gastroduodenal lesions associated with positive histology for *Helicobacter pylori* in Sikasso Hospital. A total of 34 patients were collated over a 17-month period. The average age was 38 ± 17 years and the male/female sex ratio was 2.09. In the study reported by Abdoul *et al*. and Kim *et al*. average ages were much higher than ours [2,3]. In our study, this apparently high average age could be explained, on one hand, by the insufficiency or even the absence of early follow-up of patients who are unaware of their cirrhotic status, most often revealed at the decompensated stage, but also on anther hand, by the low level of education of the population in general. The sex ratio between men and women of 2.09 is close to those reported by Calvet *et al*. [4] and Kim *et al*. [3] which were 2 and 3.9 respectively but significantly higher than that found by Abdoul *et al*. [2]. The gastroduodenal lesions represented the most frequent lesions of our endoscopic observations (33%). These results corroborate most of the studies carried out in Africa and the West [5,6], but the peculiarity in Africa is that *Helicobacter pylori* infection is very frequently associated with these digestive lesions in cirrhotic patients as evidenced by our data (57%).

In our context, this high prevalence of *Helicobacter pylori* in gastroduodenal lesions in cirrhotic can be explained by the low income of the population (poverty) in general, which is a factor favoring infection with *Helicobacter pylori*. Literature also reports that, in general, this prevalence of *Helicobacter pylori* in gastroduodenal lesions in cirrhotic patients varies according to socioeconomic level [7]. However, our results on the *Helicobacter pylori* infection rate (57%) are lower than those found in a Malagasy study [8]. In clinical terms, epigastralgia was the most common reason for consultation as reported by some authors [2,9]. Upper gastrointestinal bleeding was observed in three out of thirty-four patients and is one of the most common complications in cirrhotic patients. Literature reports that these hemorrhages are 4 to 10 times more frequent in patients infected with *Helicobacter pylori* compared to uninfected patients, whereas in the western countries, non-steroidal anti-inflammatory drugs (NSAIDs) are the most common causes of gastroduodenal ulcers (UGD) [10,11] because they are prescribed for low-dose long-term use in the majority of the population because of advanced age-related factors [12]. In our series, the gastrointestinal hemorrhage observed in the patients is explained on the one hand by the ulcerous lesions associated with portal hypertension gastropathy but also by the gastric infection with *Helicobacter pylori*, an aggression factor in the pathogenesis of the gastric lesions in an already weakened terrain by cirrhosis. Esophageal varices did not show signs of obvious bleeding apart from the red signs observed in two cases.

## Conclusion

This study reveals a fairly high incidence of *Helicobacter pylori* in the digestive lesions observed in cirrhotic patients. *Helicobacter pylori* infection in cirrhotic terrain requires urgent therapeutic management to prevent hemorrhagic complications due to the risk of compromising the vital prognosis in the short and medium term. It also reveals a high frequency of gastroduodenal lesions in cirrhotic patients since one in three patients included suffered from these lesions.

### What is known about this topic

- Literature reports that upper gastrointestinal bleeding is one of the most dangerous complications in cirrhotic patients. It also reports that the presence of Helicobacter pylori contributes significantly to the genesis of peptic ulcer disease, although its role in ulcer pathogenesis in cirrhotic patients has not yet been well established;
- Upper endoscopy is known as one of the most effective diagnostic methods for gastroduodenal lesions.

### What this study adds

- Gastroduodenal lesions in upper gastrointestinal endoscopy associated with positive Helicobacter pylori histology in cirrhotic patients is the first study conducted at the regional level in Mali;
- It allows us to provide basic data to help enrich literature but also to alert health facilities to manage these cases based on the magnitude of the problem and to improve the effective monitoring of cirrhotic patients.
